# Key role of the default mode network in transfer of motor learning from previous experience

**DOI:** 10.1371/journal.pbio.3003310

**Published:** 2025-08-15

**Authors:** Hiroshi Imamizu, Toshiyuki Kondo

**Affiliations:** 1 Department of Psychology, Graduate School of Humanities and Sociology, The University of Tokyo, Tokyo, Japan; 2 Department of Cognitive Neuroscience, Cognitive Mechanisms Laboratories, Advanced Telecommunications Research Institute International, Kyoto, Japan; 3 Department of Computer and Information Sciences, Tokyo University of Agriculture and Technology, Tokyo, Japan

## Abstract

The ability to apply skills gained in one context to different situations enables efficient learning even from limited data. This Primer explores a new PLOS Biology study which shows that the default mode network, a core brain network, plays a key role in this ability.

## Teaser

The ability to apply skills gained in one context to different situations enables efficient learning, even from limited data. A new study in *PLOS Biology* suggests that the default mode network, a core brain network, plays a key role in this ability.

Artificial intelligence (AI) and machine learning have recently achieved remarkable progress, but these systems usually need large amounts of data to learn well. In contrast, humans can learn effectively even from limited experience. One reason for this efficiency is thought to be the ability to “transfer learning”—that is, to apply knowledge or skills learned in one situation to new and different ones. This ability is especially important in motor learning, where mistakes like collisions or falls can be dangerous or costly. To make learning more efficient, it is important to use past experiences. In recent years, transfer learning has received considerable attention in the field of machine learning. This method involves reusing existing feature extractors or parts of pretrained models for new tasks. A key to effective transfer learning is accurately evaluating whether the knowledge gained in one context can be successfully applied to another. In a recent study, Rezaei and colleagues [1] proposed that the default mode network (DMN), a core of the brain’s intrinsic connectivity system, may help support this kind of transfer.

Intermanual transfer—where learning a motor task with one hand enhances performance with the other—is one of the earliest forms of learning transfer to be investigated. In experimental psychology, a classic method for studying this phenomenon involves the mirror-drawing task, in which participants trace the outline of a star while viewing only their hand’s reflection in a mirror [2]. Since its introduction, intermanual transfer has been examined in various motor learning paradigms, including sequence learning and sensory-motor tasks, where participants generate motor responses based on perceived sensory input. In neuroscience, researchers have explored the underlying mechanisms of this transfer. Key questions include whether motor skills are communicated across hemispheres via the corpus callosum (e.g., [3]), and how the type of skill—whether it is effector-dependent or effector-independent—affects the extent of transfer (e.g., [4]). These investigations often focus on motor-related brain regions and higher-order association areas, particularly the parietal and frontal cortices [5].

**Fig 1:**
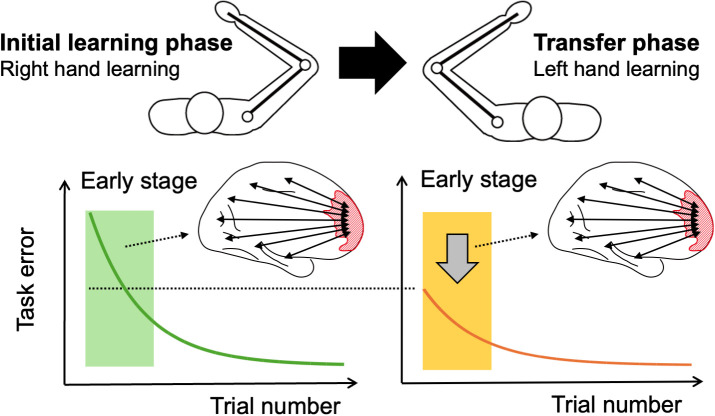
Intermanual transfer and the default mode network. In a study by Rezaei and colleagues [1], participants initially learned a sensorimotor task with their right hand (the initial learning phase). When they later performed the same task with their left hand (the transfer phase), they made fewer errors at first. Rezaei and colleagues found that brain connectivity patterns, especially those linked to the part of the default mode network (the red region), were notably similar during the early stages of the initial learning (green shaded area) and transfer (orange shaded region) phases. The brain image shows the medial surface of the left hemisphere.

Rather than limiting their analysis to motor-related regions, Rezaei and colleagues examined how functional connectivity patterns across the entire brain change during the initial learning phase (right hand) and the transfer phase (left hand) (see Fig 1). They divided the brain into over 400 regions, resulting in approximately 80,000 connections between them. To manage this complexity, they applied principal component analysis to reduce the data to a lower-dimensional space. This low-dimensional representation, known as a neural manifold, enables researchers to visualize and track large-scale brain dynamics over time (see [6] for a thorough review). This approach is particularly useful for exploratory data analysis and has recently helped generate new hypotheses in systems neuroscience. Rezaei and colleagues examined how connectivity profiles of different brain regions shifted within this low-dimensional space across various stages of learning. They discovered that regions within the DMN occupied consistent and nearly identical positions in this space during the early stages of the initial learning phase (green shaded area in Fig 1) and the transfer phase (orange shaded area). This contrasted with their observation in the motor-related region, which instead occupied different positions in the manifold space depending on which hand was being used for the task. These results suggest that DMN-related connectivity patterns established during the initial learning phase were reactivated during the transfer phase.

The DMN, originally recognized as a set of brain regions active during rest or ‘idling’, has since been revealed to occupy a core position in large-scale brain networks, integrating various information within the brain [7,8]. For instance, the DMN is involved in long-timescale information integration during narrative processing [9], and the posterior part of the DMN is known to support the retrieval of episodic memories [10]. Based on these findings, the authors propose that the DMN may contribute to intermanual transfer by evaluating the contextual similarity between the initial learning and transfer phases, and by retrieving relevant strategies from memory. Their results offer a novel perspective on how the DMN, which has not previously been well studied in the context of motor learning, might play an active role in learning transfer.

Using advanced network analysis, Rezaei and colleagues demonstrated remarkable similarity in connectivity patterns between the DMN and other brain regions during the early stages of initial learning and transfer. However, their interpretation of the functional role of the DMN seems to be largely based on the anatomical properties of the DMN as *trans-*modal cortices, as well as on analogies to previous studies on episodic memory. Future research will need to clarify the types of information re-expressed within the DMN during the transfer phase and the ways in which DMN signals influence other brain areas, especially those involved in motor control. It would also be valuable to explore whether similar mechanisms support other types of transfer and generalization of motor skills in different contexts, for example, changes in environment or tools.

While generative AI has made impressive progress in areas like language and image processing through deep learning, teaching physical agents—such as robots—to move like humans in the real world remains a major challenge. These agents must learn from data that closely depends on their own body and the environment around them. As a result, it is much harder to build large datasets for motor learning compared to those available for language or vision tasks. That is why it is important to develop systems that can learn effectively from just a small number of examples by reusing their past experiences. Understanding the key role of the DMN in how the brain learns may offer valuable ideas for designing such systems.

## Abbreviations

AI: artificial intelligence
DMN: default mode network
